# Software development process of Neotree - a data capture and decision support system to improve newborn healthcare in low-resource settings

**DOI:** 10.12688/wellcomeopenres.18423.1

**Published:** 2022-12-19

**Authors:** Nushrat Khan, Caroline Crehan, Tim Hull-Bailey, Charles Normand, Leyla Larsson, Deliwe Nkhoma, Tarisai Chiyaka, Felicity Fitzgerald, Erin Kesler, Hannah Gannon, Patty Kostkova, Emma Wilson, Matteo Giaccone, Danie Krige, Morris Baradza, Daniel Silksmith, Samuel Neal, Simbarashe Chimhuya, Msandeni Chiume, Yali Sassoon, Michelle Heys

**Affiliations:** 1 Population, Policy and Practice, UCL Great Ormond Street Institute of Child Health, London, WC1N 1EH, UK; 2 Neotree Charity, London, EC2A 2RS, UK; 3 Independent contributor, London, UK; 4 Biomedical Research and Training Institute (BRTI), Harare, Zimbabwe; 5 Kamuzu Central Hospital, Lilongwe, Malawi; 6 Department of Infectious Disease, Imperial College London, London, UK; 7 Children's Hospital of Philadelphia, Philadelphia, USA; 8 UCL Centre for Digital Public Health in Emergencies, London, UK; 9 Baobab Web Services, City of Cape Town, South Africa; 10 University of Zimbabwe, Harare, Zimbabwe; 11 Snowplow Analytics, London, UK

**Keywords:** Digital health system, neonatal health support, user-centred design

## Abstract

The global priority of improving neonatal survival could be tackled through the universal implementation of cost-effective maternal and newborn health interventions. Despite 90% of neonatal deaths occurring in low-resource settings, very few evidence-based digital health interventions exist to assist healthcare professionals in clinical decision-making in these settings. To bridge this gap, Neotree was co-developed through an iterative, user-centered design approach in collaboration with healthcare professionals in the UK, Bangladesh, Malawi, and Zimbabwe. It addresses a broad range of neonatal clinical diagnoses and healthcare indicators as opposed to being limited to specific conditions and follows national and international guidelines for newborn care. This digital health intervention includes a mobile application (app) which is designed to be used by healthcare professionals at the bedside. The app enables real-time data capture and provides education in newborn care and clinical decision support *via* integrated clinical management algorithms. Comprehensive routine patient data are prospectively collected regarding each newborn, as well as maternal data and blood test results, which are used to inform clinical decision making at the bedside. Data dashboards provide healthcare professionals and hospital management a near real-time overview of patient statistics that can be used for healthcare quality improvement purposes. To enable this workflow, the Neotree web editor allows fine-grained customization of the mobile app. The data pipeline manages data flow from the app to secure databases and then to the dashboard. Implemented in three hospitals in two countries so far, Neotree has captured routine data and supported the care of over 21,000 babies and has been used by over 450 healthcare professionals. All code and documentation are open source, allowing adoption and adaptation by clinicians, researchers, and developers.

## Introduction

Nearly 80% of births in low-resource settings (LRS) now occur in healthcare facilities ( World Health Organization, 2020a). Yet neonatal mortality rates remain persistently high and progress on newborn mortality lags behind reductions in maternal and infant mortality. Each year 2.4 million babies die in the first 28 days of life, 90% of whom die in low and middle-income countries ( World Health Organization, 2020b). There are also stark disparities in newborn survival rates. Sub-Saharan Africa has the highest newborn mortality rate, where a child is 10 times more likely to die than those born in high-resource settings. This is followed by central and southern Asia ( World Health Organization, 2022).

The United Nations’ Sustainable Development Goal (SDG) target is to reduce neonatal mortality to less than 12 per 1000 live births by 2030 ( Golding *et al.,* 2017). To accelerate progress in the regions yet to achieve this goal, it is critical to understand the underlying clinical and contextual causes of newborn deaths. Implementation of low-cost evidence-based interventions can help characterise these causes early and tackle two-thirds of newborn deaths in a systematic manner ( Bhutta *et al.,* 2014). However, detailed, individual-level data from healthcare facilities are rare. Very few evidence-based digital health interventions exist in LRS, while national electronic health record (EHR) systems are underdeveloped and rarely include neonatal modules ( Stevenson *et al.,* 2021). National data from LRS are available for a few specific interventions or disease cohorts only. For example, the Vermont Oxford Network (VON) supports collection and analysis of facility-based neonatal care data *via* a paid membership model, where data are entered retrospectively into the database from clinical records by data clerks. This means most member hospitals are from high income countries except Ethiopia, which uses a separate model as a result of a decade-long partnership with VON ( Edwards *et al.,* 2019).

Other types of mobile health interventions include clinical decision support (CDS) tools containing algorithms and reminders, digital guideline apps and serious mobile games. For example, the mPneumonia app in Ghana combined CDS to prompt healthcare professionals (HCPs) in the management of pneumonia according to current guidelines ( Ginsburg *et al.,* 2015). However, it is a vertical tool, focusing on one disease only. An example of a serious mobile game intervention is the Maternal and Neonatal Technologies in Rural Areas (MANTRA) project which used serious games aimed at low-literacy women in rural Nepal. Even though this intervention demonstrated significant knowledge gain of the participants, it did not focus on improving maternal and newborn care in healthcare settings ( Mueller *et al.,* 2020).

To address this need to collect reliable data and standardise newborn care in LRS, Neotree was co-developed in collaboration with HCPs in the UK, Bangladesh, Zimbabwe, and Malawi. The Neotree platform uses a mobile application to perform prospective data capture and provide clinical decision support and education in newborn care ( Heys *et al.,* 2022), which falls within tier C of digital health technologies’ classification published by the National Institute of Health and Care Excellence ( NICE, 2018). This low-cost digital health system is used to improve postnatal care at the bedside in LRS and to provide insights into population health trends. The main Neotree database consists of data collected by HCPs at the bedside as an integral part of the admission and discharge process in neonatal intensive care units (NICU), and these data are used to inform clinical decision making, *e.g.,* quality improvement projects, development of in-app algorithms.

At the time of initial Neotree conception in 2013, few applications existed that were integrated within newborn clinical care. Some applications under development were being designed for HCP training rather than bedside care. For example, AIIMS-WHO CC Standard Treatment Protocols (STP) is an application for newborn management that was developed by All India Institute of Medical Sciences (AIIMS) around the time when Neotree was conceptualised ( Prakash *et al.,* 2016). Similarly, the Safe Delivery App was developed in collaboration with the Universities of Copenhagen and Southern Denmark, which is designed to provide training on obstetric and neonatal emergencies ( Lund *et al.,* 2016). But neither of these examples include data capture or patient specific clinical decision support. Furthermore, besides being training focused, the proprietary nature of these software means they cannot be easily adapted according to country and site-based needs. 

More recently researchers have focused on the development of standalone digital CDS, some of which are linked to educational tools. For example, Noviguide, a proprietary software application, has been developed over the last few years to provide CDS for some common newborn conditions such as neonatal sepsis ( Muhindo *et al.,* 2021). Crucially however, these CDS tools are not integrated with routine data capture functionalities and EHR for newborn babies ( Bucher *et al.,* 2020; Chan *et al.,* 2021; Muhindo *et al.,* 2021; Schaeffer *et al.,* 2019). As a result, these apps often rely on voluntary use of CDS tools, assuming a level of pre-use knowledge, *e.g.,* the HCP has to suspect a specific condition is present in order to access the guide in how to manage it. This can lead to low adoption rates, for example, in a pilot study using NoviGuide in Uganda, 36.25% (1,705 out of 4,704) admissions were entered by HCPs in the app ( Muhindo *et al.,* 2021).

Continuous development of Neotree over the past eight years and implementation in two countries means that the Neotree system is refined, equipped with complex clinical algorithms for critical decision-making and adaptable in different LRS. As of July 2022, the Neotree has collected routine data from, and supported the care of over 21,000 babies admitted to three hospitals - two in Zimbabwe (Sally Mugabe Central Hospital (SMCH) in Harare and Chinhoyi Provincial Hospital (CPH) in Chinhoyi) and one in Malawi (Kamuzu Central Hospital (KCH) in Lilongwe) - the largest known detailed database on neonates from low-resource countries. These data have enabled clinicians to monitor admission, mortality and morbidity trends and assess quality improvement interventions ( Chimhuya *et al.,* 2022).

## Methods

**Figure 1.**
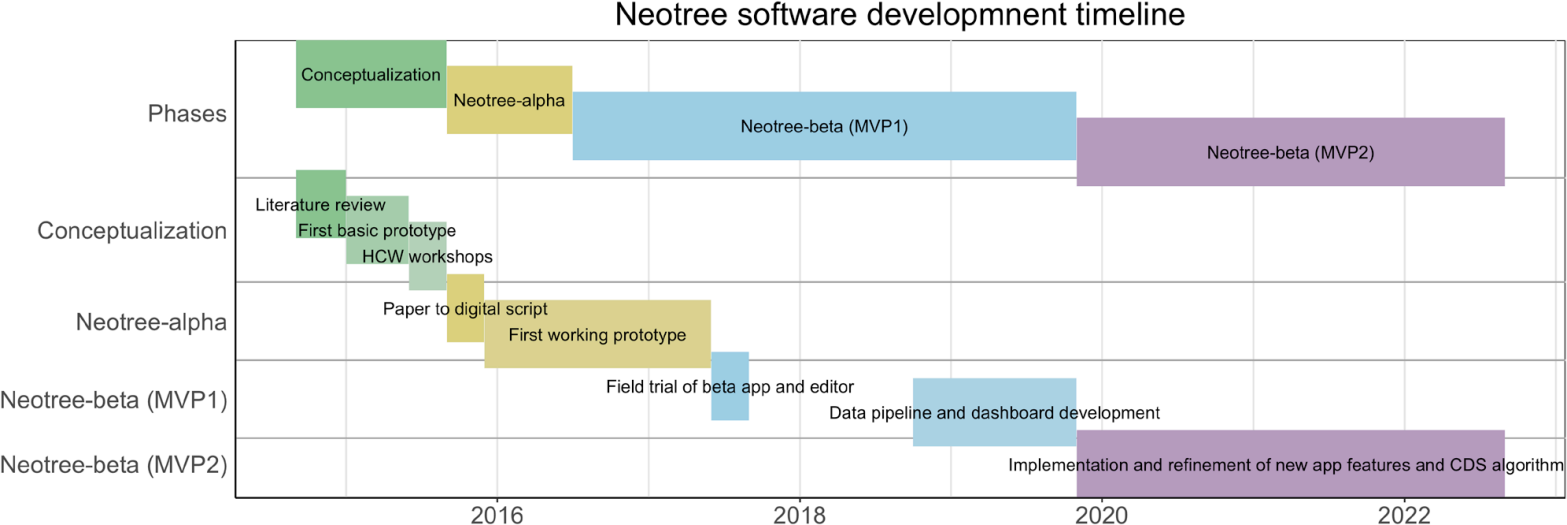
Neotree development timeline.

The Neotree ( Khan *et al.* 2022) was developed in a user-centric iterative approach while addressing the gaps in LRS and needs of HCPs. The team followed the Medical Research Council guidance for developing and evaluating complex interventions and agile software development cycle that incorporates incremental and iterative development ( Agarwal *et al.,* 2016; Campbell *et al.,* 2000; Leau *et al.,* 2012). Within implementation we have taken into account country-specific needs and adaptation for National level EHRs. In this paper we will describe these iterative development phases within each component of the Neotree system ( Figure 1). First, we describe conceptualization, then pre-prototype, followed by prototype development, and finally piloting and implementation. Findings from mixed methods evaluation of the Neotree system are described elsewhere ( *e.g.,* Crehan *et al.,* 2019; Gannon *et al.,* 2021; Heys *et al.,* 2022) and other related data analysis are ongoing ( Wilson *et al.,* 2022).

### Conceptualization

A literature review of HCP-led newborn interventions to improve outcomes for low birthweight babies in lower-income countries identified gaps in successful implementation of proven interventions ( Kesler, 2013). In other words, even though evidence-based interventions existed, these were not being used in standard clinical care at that time. Therefore, it was hypothesised that reductions in newborn mortality could be achieved through equitable and sustained improvements in the quality of newborn care through ensuring uptake of evidence-based and standard newborn care in health facilities ( Bhutta *et al.,* 2014). The assumption was that a digital system that captured clinical data at the point of care and then used these data to (a) trigger both educational messages about how to deliver quality newborn care ( *e.g.,* how to check a babies temperature and why this is important) and (b) when and how to provide best evidenced clinical care ( *e.g.,* when and how to keep a newborn warm) would result in sustained uptake of evidence based interventions and thus improved care and survival.

### Pre-prototype

Authors collaborated with the UCL Computer science team to develop a basic android app prototype for data capture and clinical decision support in 2014, including an exemplar clinical care pathway for thermoregulation of the newborn ( Kesler, 2013). PowerPoint (Microsoft, 2022) images of the prototype were presented to a workshop of HCPs in Bangladesh ( *n*=15), who found the concept of device-enabled decision support to be acceptable, but also emphasised any such tool would need to address the clinical needs of all admitted babies, irrespective of weight, and would need to be configurable to the level of care deliverable in a given health facility ( *unpublished*). In response to this feedback, subsequent development focused on the care of all babies and the need to be adaptable to different settings.

### Prototype development

**Figure 2.**
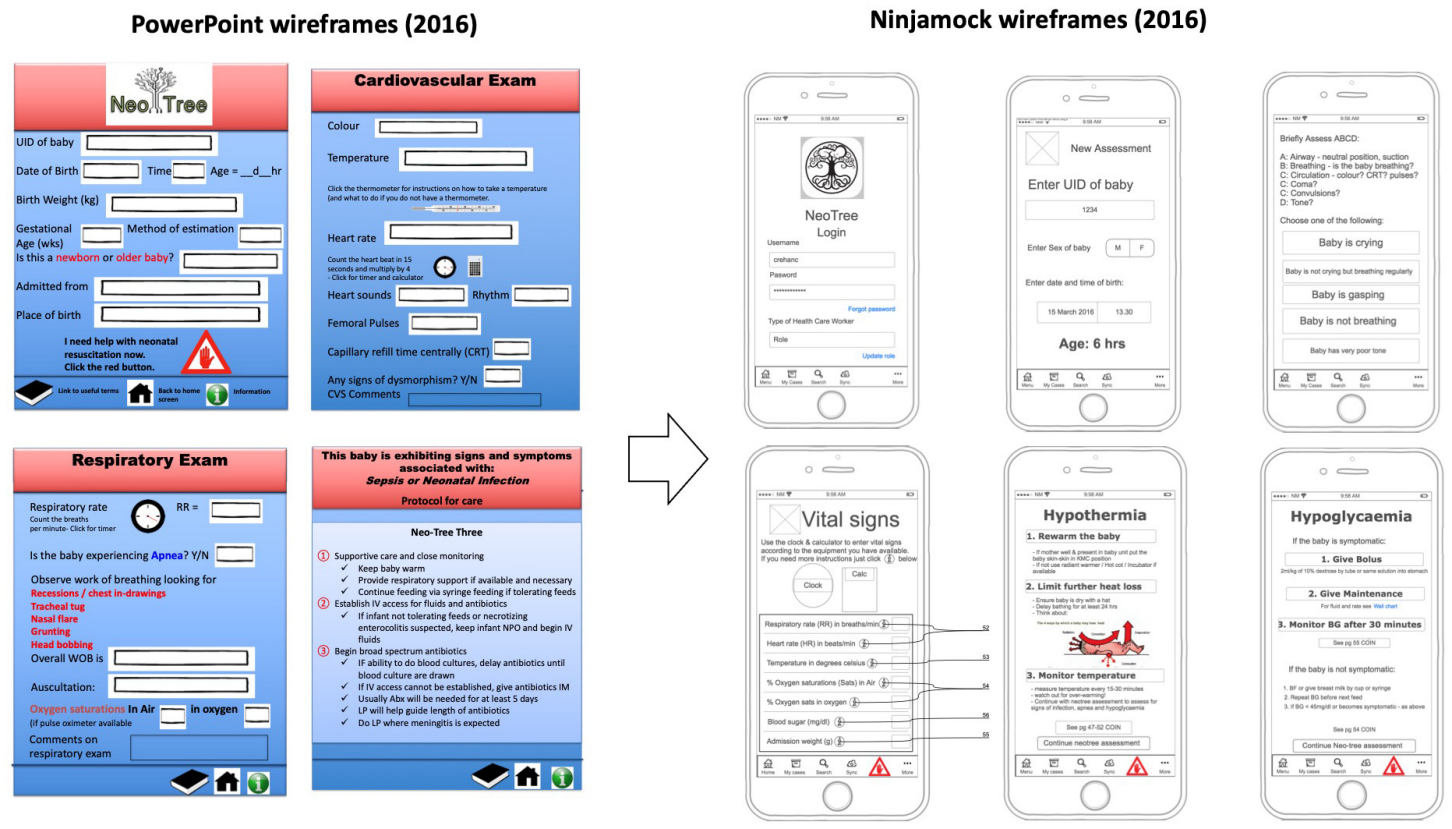
Prototype wireframes.

Following the conceptualization phase, the idea of building the Neotree app interface and algorithm around the structure of the existing paper neonatal admission form was introduced and the first working prototype of Neotree was created in 2016 ( Crehan *et al,* 2019). This consisted of three components: 1. An android mobile and tablet app, 2. A web editor that allows a non-developer or clinician to edit and customise the app interface, and 3. Google Firebase framework, where any configuration changes were automatically propagated to mobiles and tablets with the app installed (given they had internet connectivity and the app was loaded). Wireframes of the app were at first drawn up in PowerPoint (version 16.0) and then using a proprietary software, Ninjamock to communicate the desired design of the user interface (UI) to the developer with the help of a user experience expert ( Figure 2). The development and use of the system, then leveraged a design system built in a mock up prototype tool, InVision with user experience expert assistance. This enabled the developer to build up the UI code efficiently. 

### Piloting and implementation

Using the first web-editor and Google Firebase framework, a working prototype of the Neotree app (Neotree-Alpha) was translated into practice and evaluated over a one-month pilot at Zomba Central Hospital (ZCH), Malawi ( Crehan *et al.,* 2019). Data collected during this period were stored at the hospital on a study laptop and then manually transferred. Using this data, the concept of developing a data dashboard was tested using Microsoft Excel (version 16.0, RRID:SCR_016137) ( Crehan *et al.,* 2020).

Later during an implementation evaluation of Neotree at KCH between May and October 2019, a data pipeline consisting of an application programme interface (API) for secure data transfer and a database for data secure storage hosted in Amazon Web Services ( AWS, 2022) was added. As a result, an evolving cloud hosted data visualisation dashboard was built and piloted using Microsoft Power BI ( Microsoft Power BI, 2022). It supported data cleaning, transformation and visualisations incorporating behavioural sciences theory and was hosted on Microsoft Office365 ( Crehan, 2022). 

Below we describe the four key components of the Neotree system that evolved based on user needs and iterative testing of the platform: 1. The Neotree web editor, 2. The Neotree mobile app, 3. The Neotree data pipeline, and 4. Data dashboard.

**Figure 3.**
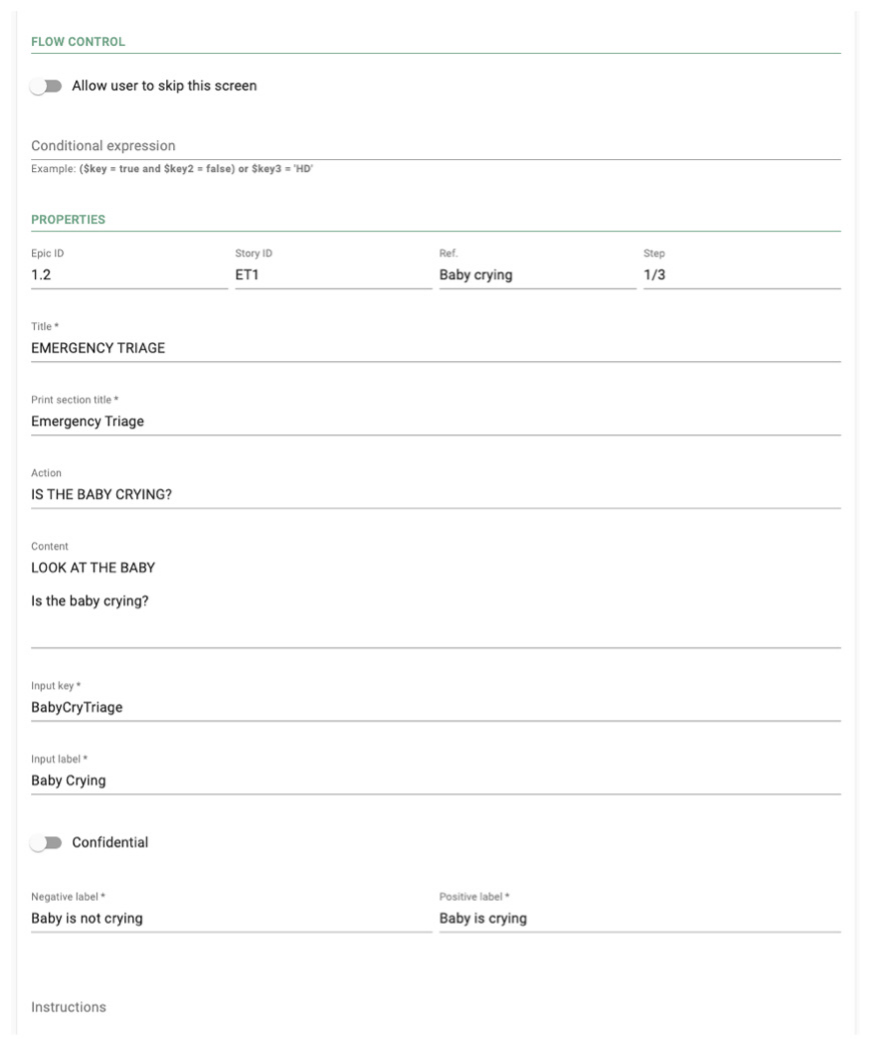
A sample page of the Neotree web editor.

***The Neotree web editor.*** The web editor enables HCPs to create "scripts", where each script defines a specific workflow in the mobile app, *e.g.,* a workflow to follow when a baby is admitted, and another when a baby is discharged ( Figure 3). Each script consists of an ordered list of configurable screens devised by a scripting engine, *i.e.*, in the web editor it is possible to specify exactly what is shown in the screen, what data is collected, and what conditions need to be true for the screen to show. For example, a screen might prompt a HCP to enter some data, perform a check or administer some treatment ( *e.g.,* "warm newborn") or suggest a diagnosis.

The configuration of each script is saved to the Neotree backend. Scripts are translated and rendered by the mobile/tablet application code and automatically reflected in near real-time to the app interface. The mobile application will, if it senses a network connection, check to see if a new version of the script is available and download it locally. It will then display the correct screens in the correct order, as specified by the script.

During pilot implementation of Neotree at ZCH, new essential features required for building an interactive newborn admission form were added iteratively to the web editor, as well as to the mobile or tablet app rendering code, *e.g.,* the ability to add pictures to a form ( Crehan *et al.,* 2019). In this way the flexibility of the editor app allowed users to prioritise their needs during early development so that the features most important to them (both clinically and for ease of workflow) could be executed in the app in the first few weeks of piloting, thus promoting local ownership and buy-in.

***The Neotree mobile app evolution.*** The next significant release, Neotree-Beta (MVP-1) was co-developed with Malawian HCPs ( *n*=46) in 2016-17 ( Crehan *et al.,* 2019 & Crehan *et al.,* 2020), focusing on configuration refinements using the editor to incorporate the detailed feedback of nurses using the app for the first time in the ward. The application continued to be executed on Android tablets and mobile phones in the neonatal wards, with HCPs capturing patient data and guiding clinical practice when patients were admitted and discharged, including the recording of blood test results from the labs and perinatal data during pilot implementation (2018 onwards) at SMCH in Zimbabwe ( Gannon *et al.,* 2021). During this time, the web editor continued to show value in the way scripts could be quickly authored and adapted in different environments, *e.g.,* adding new data fields, editing or deleting existing data fields *etc*. The app continued to enable operation in a low or occasional connectivity setting: connectivity only being required to sync forms (for example, if an admission or discharge form has been updated) and submit data to the backend or receive new configuration information. This is an important and essential feature to implement this type of healthcare system in an LRS. Further user testing at KCH in Malawi led to significant refinement of the user interface based on user feedback, such as improved navigation, data validation for certain fields and enhancing efficiency of data entry by automating calculations. These changes were applied to the consecutive release of Neotree-Beta (MVP-2).

***The Neotree data pipeline.*** The data pipeline manages data flow from the app to the backend and then to data visualisation dashboards for analytics purposes. The Neotree backend houses two types of information: 1. The data captured from the Neotree mobile application, and 2. The configuration scripts used to determine the different workflows or scripts as mentioned in the Neotree web editor section above. Subsequent evolution of the app retired the use of the Firebase framework and employed the database. The representational state transfer (REST) API enables the web editor to publish updated versions of scripts to the backend, and a route for the mobile applications to fetch the latest script. It also provides a write API for the mobile apps to publish collected data.

The prototype implementation of data transformation in Power BI was replaced by the data pipeline built in Python (using Kedro as a data processing framework). It processes the raw data in JSON format from the database and reformats and reshapes them into tables that are easier to analyse and visualise in the data dashboard by staff in hospitals and can be used for further exploration of data. The process also involves cleaning and deduplication of the records in cases where multiple instances of the same record have been exported. To maintain data integrity, the data from the tablets are saved into their own schema where no alterations are made, *i.e.*, in their raw state, then a cron job will run periodically generating derived tables which are then used for visualisation and further analysis.

***Data dashboard.*** At first Power BI was selected as the first data visualization tool to create a dashboard for reporting and quality improvement purposes in the hospital and tested in Malawi ( Crehan, 2022; Mgusha *et al.,* 2021). After this testing phase, Power BI was replaced by a non-proprietary and simpler tool, Metabase (2022) in the production phase for larger scale implementation as it is more feasible to use in an LRS where internet connectivity can be less reliable. It similarly empowers HCPs to easily interrogate the data and create visualizations *via* a web application to be displayed in the hospital wards.

**Figure 4.**
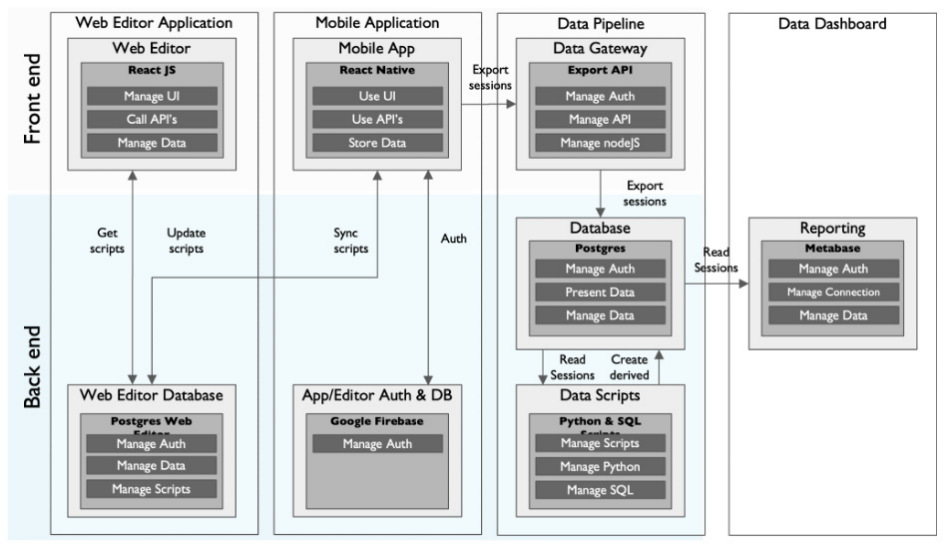
Neotree system architecture.

Figure 4 below demonstrates how different components of the Neotree platform work together for its functionality.

*Operation*

**Figure 5.**
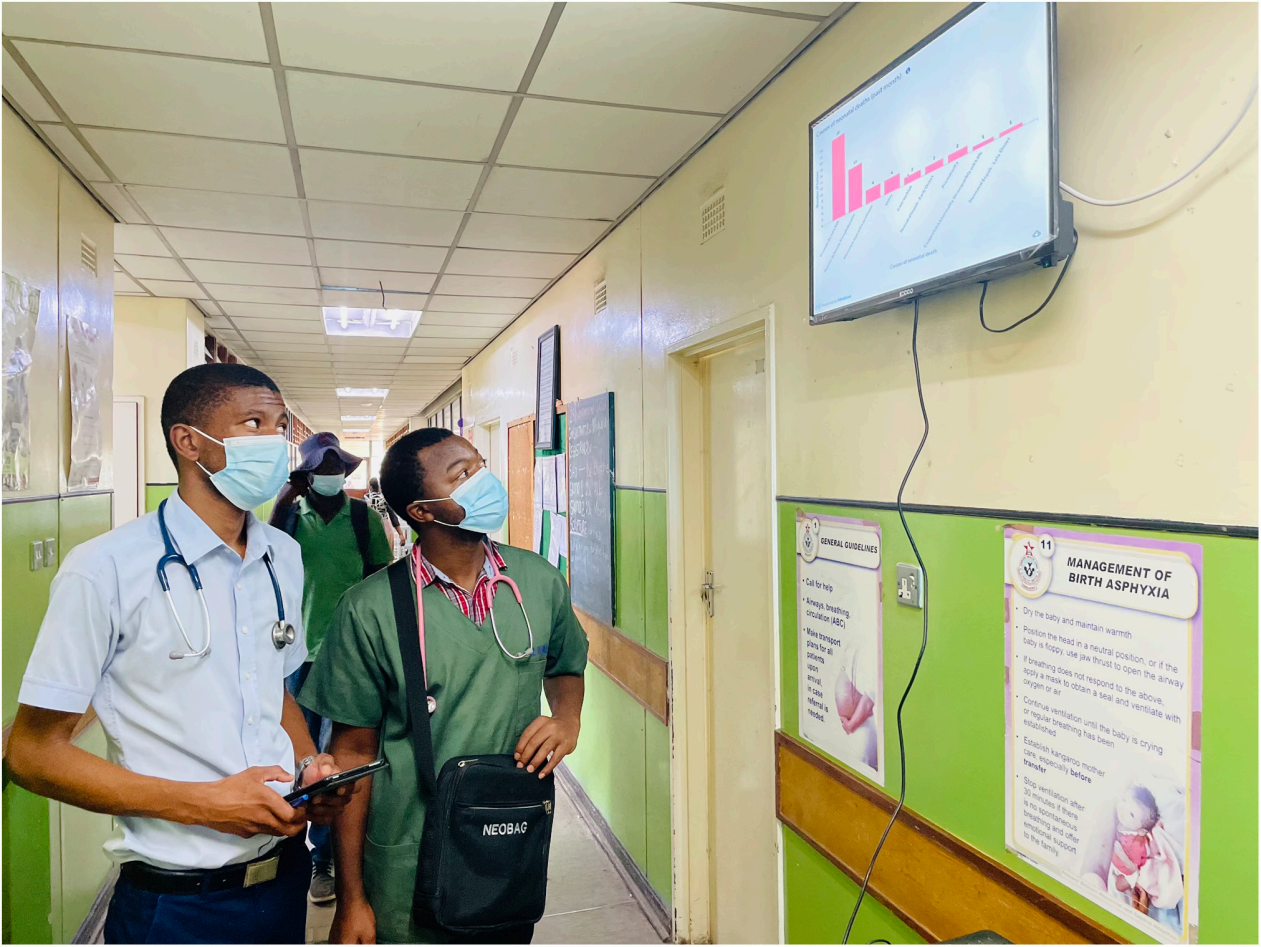
Data dashboard implemented at Sally Mugabe Central Hospital in Zimbabwe.

Implementation requirements of the Neotree system are the following:

- The mobile application was developed using React Native (version 0.66.0) and requires a tablet or mobile phone to run Android 11 or newer Android versions.
- The application fetches configuration information from the Neotree backend *via* a RESTful API. The Neotree backend is a node.js application (developed using node.js version 12.19.0) backed by Postgres (version 10.18). The backend manages script configuration and storage and serving of data to and from the mobile application. It is set up on a secure server running on a Linux server (Ubuntu 18.04).
- The web application, running on Nginx (1.14.0), managing the script configuration is hosted on the same server that runs the backend. This is an application built using React (version 17.0.2). It provides HCPs with an easy-to-use user interface to configure the scripts. Scripts are versioned, making it possible to roll back updates to scripts, and distinguish data collected against different script versions.
- The mobile application submits data collected to the backend *via* the API in a JSON format. A Python data pipeline, setup to run regularly at a configurable frequency ( *e.g.,* hourly), processes the data, taking the JSON in Postgres and shredding it into other tables in Postgres that are optimized for analytics (One column per data point). The data pipeline has been built using Python (version 3.6.9) and Kedro (version 0.17.0).
- Metabase (0.41.4) has been installed on the same server that runs the web editor, Neotree backend and data pipeline. This has access to the output of the data pipeline (that is saved in derived tables in Postgres) and provides a secure way for HCPs to access, query and visualise the data.
- Dashboards built using Metabase are then displayed in the wards and outcomes are communicated with HCPs in the implementation and control sites ( Figure 5). Because internet connectivity in the ward for accessing Metabase dashboards live cannot be relied on, compressed image exports of the dashboards are created frequently (every three hours) on the Neotree server. A Raspberry Pi (model 3B+) in the ward fitted with a SIM card (GSM SIM 868 HAT, 3G/4G) is configured to regularly fetch the dashboard images from the server and display them on a monitor in the ward. The entire Raspbian operating system was used for the dashboard, which was known as Raspbian Buster at the time. Raspbian is a Debian Linux distribution, designed specifically for Raspberry Pi. The software is open source and can be run on any commodity computers running Linux or Windows operating system.

*Usability testing*

We have worked closely with HCPs using Neotree to (i) understand and test the changes made by each release on the usage and (ii) understand satisfaction levels with the core features of Neotree ( Wilson *et al.,* 2022). User feedback has been collected in mainly three ways:

(i) On an ongoing basis: This includes facilitating open communication with the HCPs using the application, allowing them to provide feedback on specific aspects. Furthermore, data collected using Neotree are discussed at the weekly educational meetings led by the paediatric consultant to gather feedback and suggestions. These feedbacks are then used to drive the iterative development of the platform

(ii) During usability workshops: These workshops were conducted with the release of significant new functionality, *e.g.,* the release of the dashboards and the diagnostics functionality, and were administered by a trained human-factors engineer. Between January and May 2022 for example, sessions were run following each major diagnostic functionality rollout in SMCH, involving five HCPs at each stage and lasting approximately 45 minutes. This involved HCPs completing an ‘admissions’ script using a predetermined use test case. Recorded and transcribed sessions afterwards allowed for behavioural observations to understand the human factors of the device and probing with follow-up questions based on HCP actions.

(iii) Using specific usability questionnaires such as the Systems Usability Scale (SUS) and the Post-Study System Usability Questionnaire (PSSUQ) ( Wilson *et al.,* 2022), which were conducted in June 2022 at SMCH to quantify the user experience of the Neotree ( *n*=10).

*Diagnostic functionality*

**Figure 6.**
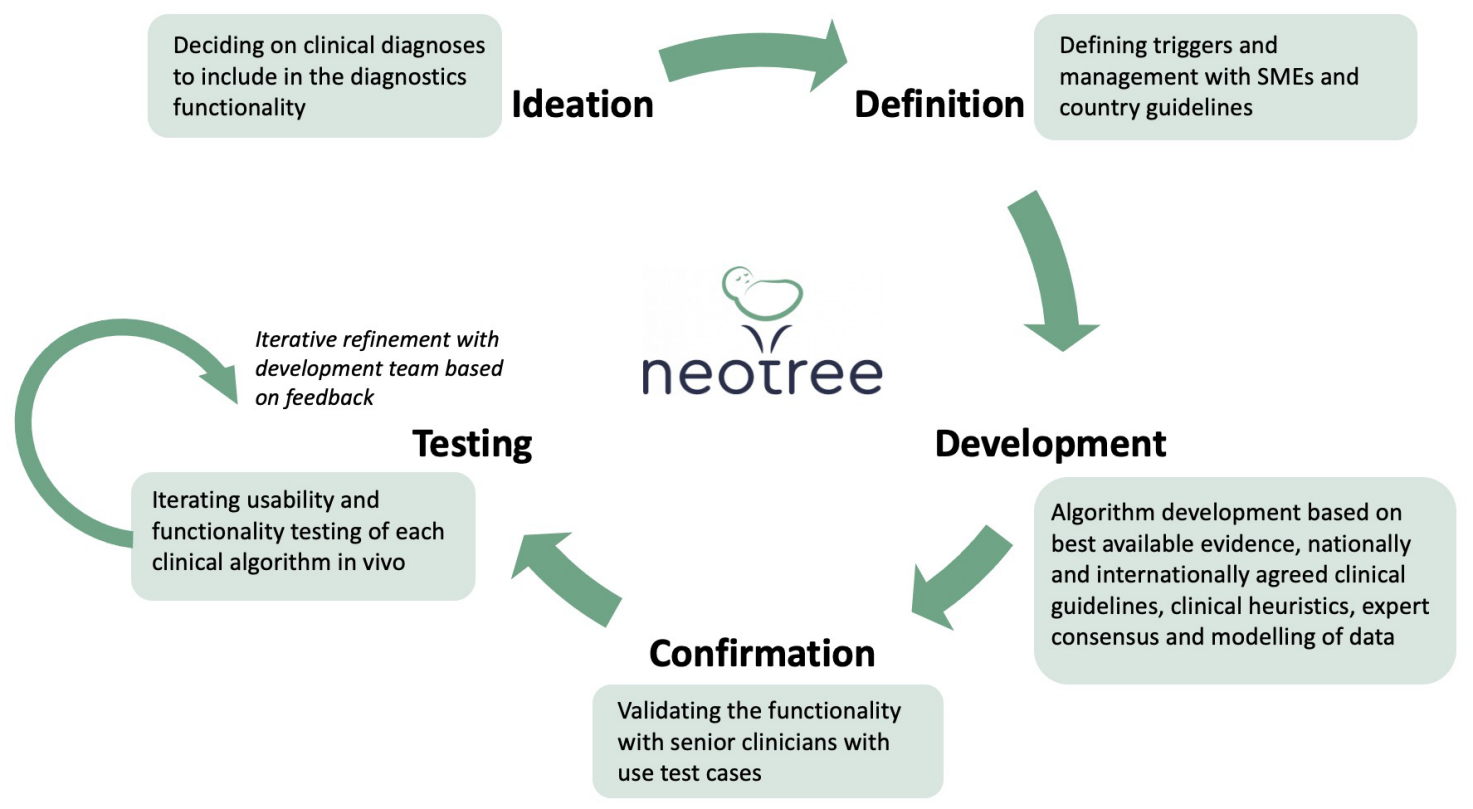
Development process of diagnostic functionalities.

One of the implementation goals for Neotree was to integrate a “clinical diagnosis and management support functionality” as part of the admission process. This functionality essentially provides the HCPs with “suggested” diagnoses based on entered clinical data. It allows the HCPs to first select their own diagnoses from a list of diagnoses and then provides suggestions if the algorithms generated from clinical data formed a diagnosis that was not picked by the HCP. This functionality and the specific clinical algorithms were developed in a number of phases ( Figure 6).

First, the algorithms for resuscitation and stabilisation of the sick newborn were developed in the UK during pre-prototyping and prototyping based on best available international and national guidelines. These were reviewed and confirmed by a clinical team formed by senior clinicians in the UK and Malawi ( *n*=6) prior to implementation during the ZCH pilot study in 2016. An early version of an expanded set of diagnostic algorithms addressing a wider range of neonatal conditions was also developed at this time, but not implemented within the app for clinical use as they were incomplete and required further development. Data fields for incorporation into ongoing algorithm development were based on the Malawian Ministry of Health paper admission forms, clinical experience at ZCH, local and international guidelines and in discussion with senior Malawian clinicians.

Second, the team conducted a Delphi study with international experts in newborn care to review four of the algorithms, three of which had not yet been implemented within the Neotree app: sepsis, neonatal encephalopathy and respiratory distress and one of which was already implemented as part of the resuscitation and stabilisation algorithms: thermoregulation ( Evans *et al.,* 2021). Significant changes were made to the sepsis, neonatal encephalopathy and respiratory distress algorithms and additional analysis plans were developed to address knowledge gaps in how to create the sepsis algorithm in particular. Third, whilst ongoing work to inform and strengthen these and other algorithms was underway, clinical management advice was incorporated into the app. This means when a HCP chose a possible diagnosis from a given list at the end of the admission process, they were provided with associated, evidence-based clinical management guidance.

Fourth, over a two-year period and through a repeated review of available literature ( *e.g.,* Neal, 2022) and national and international guidance, modelling of the clinical data (Neal, 2022) and joint meetings with clinical teams from the UK, Malawi and Zimbabwe, the remaining clinical algorithms were completed and incorporated into the app. An iterative process of testing, feedback, and further development followed this to optimise functionality. Mock clinical test cases created with clinicians were used for testing in the app to ensure that the algorithms are triggered in the correct circumstances. This led to refinements in the algorithms themselves and/or feedback to the software development team if the algorithms were not triggering correctly.

Once algorithms were approved, HCPs carried out previously explained usability sessions to run through the test cases providing “think aloud” feedback ( Charters, 2003) on each page of the diagnostic functionality, describing their understanding of the presented information, as well as how the information was being presented, and whether the tasks and goals were clear to them. This feedback led to further development in the content, layout, screen flow of the diagnostic functionality which was updated either by the web-editor, depending on whether it was a content update or the software development team in case a more fundamental, structural update was required.

So far, the following diagnostic algorithms have been developed and are implemented as part of daily clinical care at SMCH, CPH and KCH: resuscitation and stabilisation, thermoregulation, convulsions, low birth weight, prematurity, hypoglycaemia, HIV, respiratory distress, neonatal encephalopathy, sepsis, syphilis, jaundice and some congenital abnormalities (cleft lip/palate, congenital dislocation of the hip, talipes, gastroschisis and omphalocele, and spina bifida). Ongoing analysis of the clinical database is underway to optimise these algorithms ( *e.g.,* to create a data driven predictive model for neonatal sepsis ( Neal *et al.,* 2022)).

## Key features

A number of critical features have been built as part of the Neotree system:

- Data collection: Data are captured in real-time when HCPs admit, discharge and log laboratory records in the Neotree. To adapt to low or no internet connectivity in hospital wards in LRS, admission data can be entered into the app offline and synced with the database when internet connection is available. Only pseudonymized data are stored in the Neotree backend, excluding names and addresses. When an HCP enters a recognized pseudonymized identifier into a discharge form, any data fields that are shared between the discharge form and the corresponding admission form are pre-populated, to save the HCP time. In the event that the tablet does not have internet connectivity, data will only be pre-populated from submissions that have been made on the tablet device. With internet connectivity, prepopulation can be based on data entered into any one of the ward tablets.
- High customizability *via* a web editor: The web editor is aimed to make the development process more rapidly iterative, agile and user-centric that allows HCPs to incorporate their insights into the app with a very short iterative cadence, as well as customise the mobile app experience directly themselves, based on how the app is used and availability of local resources.
- Bedside decision support: Neotree defines a standardised workflow and examination for the HCP to follow when admitting a newborn. Educational information, guidelines and images can be included and triggered at the appropriate point at the bedside, to guide and support the HCP in examining and delivering quality newborn care. This is particularly important in an environment where HCPs receive limited formal training and often have limited experience of dealing with sick babies when they are deployed on the wards. Senior support may also be remote or difficult to access.
- Dashboard and reports: Collected data are available to query and aggregate *via* a dashboard and reporting interface and displayed in the wards, which can then be used for monitoring and quality improvement purposes by HCPs. Examples of key outputs in monthly dashboards include number of admissions, discharges, and deaths, trends in case fatality rate, comparison of case fatality rates across implementation sites, key causes of deaths, hypothermia rate *etc*. Figure 6 shows a data dashboard use case at SMCH.
- Data ownership: Neotree ensures that the Ministry of Health in each country is the owner of data collected through hospitals and data collection process is streamlined to align with country-based government data sharing policies, *e.g.,* a server was installed at SMCH in Zimbabwe to ensure collected data are stored within the country.
- Diagnostic support: Algorithms based on existing local, national and international clinical guidelines and best available evidence have been developed, tested and refined using data collected *via* Neotree. These algorithms were implemented in the app that suggest diagnoses and management for newborns based on real-time data input, providing additional support to HCPs using the app.
- Adaptability to local needs and resources: The Neotree system was built so it can be adjusted to be suitable across different settings, given there could be differences in prevalence of certain diseases, management per country guidelines, or availability of equipment. For example, neonatal sepsis is one of the key causes of newborn deaths in Zimbabwe and therefore clinicians at SMCH (which has a microbiology laboratory on site) have placed emphasis on streamlining and improving the process of collecting blood cultures and using the results ( Gannon *et al.,* 2021). Powered by blood culture data collection through a Neotree script, clinicians are now able to monitor contamination rate, learn about key organisms detected in the cultures, and then communicate the findings with HCPs for further improvement. Such rich data with linked clinical features, management decisions, laboratory results, and outcomes from low-resource countries are rare, which makes it a valuable resource to be used for predictive modelling to develop algorithms.
- Applicability in a wide range of settings: While Neotree data are beneficial to derive disease-specific solutions, the platform itself is not limited to any specific newborn conditions and thus allows a broad range of data supported, evidence-based decision-making. For example, the hospitals continued to collect data using Neotree during the pandemic and comparative analysis suggested that impact of pandemic could be context specific, while emphasising the need to collect such data at a national level ( Chimhuya *et al.,* 2022).

## Use case

### Setting up the Neotree platform

The process to set up the Neotree backend, web editor, data pipeline and the app itself can be found in the respective GitHub repositories as reported in the software availability section and outlined below. These individual repositories are linked to the central GitHub repository. This also includes instructions to set up a demo app and web editor, as well as a synthetic dataset ( Khan, 2022). A demo data dashboard built using this synthetic dataset on Metabase is available on request.

To set up the platform, at first the backend, NodeJS API is configured by setting up the local configurations in a “.env” file. This needs to be placed in the home directory of the project following the example provided in the source code’s “env-example” file. After successful setup, the backend should be started using the commands specified in the readme file here. 

The Neotree Web Editor that allows setting up a data collection form is then set up by following the configurations specified in the readme file of the source code with example configurations available in the “env-example” file ( here). The forms created on the web editor can be customised depending on the needs of a healthcare facility. Postgres databases for the web editor, and data entry sessions need to be set up before running the web editor and NodeJS API.

The mobile app is then set up by following the specified steps in the readme file of the react native app ( here). This will allow the app to connect and pull data from the web editor, to connect to the NodeJS API for data syncing and then display the forms created using the editor, as well as allow for data input. The data is stored in the data collection device’s database to allow for offline data collection and the complete data forms are synced to the main Postgres database automatically whenever the device is connected to the internet. Manual data syncing can also be triggered through the mobile app’s export functionality.

The final step is to set up and configure a data pipeline by following steps specified in the readme file of the source code ( here). Following the specified configurations, the data pipeline can be set to run automatically at configurable intervals. This is a python code base used to manipulate raw exported data from Postgres database into derived tables that are easier to ingest for business intelligence tools for data visualisation, such as Metabase. This code base should run on any operating system that supports Python, but comprehensive tests have been run on Ubuntu 18.04 and Windows 10.

### Using the Neotree platform

***Creation of the forms in the editor.*** Neotree supports the collection of a range of clinical and sociodemographic data. The link to a comprehensive and evolving data dictionary is available here.

Upon setting up the platform, users can create a data collection form on the editor that is then rendered on the mobile application. These forms are dynamic, support input of different data types and conditional statements *via* the web editor. Texts and images for clinical decision support and training are also added during form creation.

***Display of forms on mobile and data collection.*** Based on the input from the connected web editor, a list of available forms is displayed on the home screen upon logging on to the mobile application. When a user selects a form, specific questions are then displayed. A question can be mandatory or optional depending on specific conditions on the editor. At the end of each section, a progress screen indicates what portion of the form has been completed and what is still left. Users can navigate back to a specific section if needed. Upon completion of a record, the form is saved and once confirmed the record can no longer be changed. Users can view the summary of entered data and print a copy directly from the mobile application. The data is then synced automatically to the Postgres database when the mobile device gets connected to the internet. Data can be manually exported from the app in order to generate Excel and JSON files that can be used for data backup if needed.

### Visualisation of data on Metabase

Once the data has been exported to the Postgres database, the Python data pipeline will run periodically as configured, and process the data into derived tables which presents a format that is ingestible by Metabase. Metabase should be configured to point to the Postgres database containing the derived tables. This allows interrogation with the data and creation of aggregated outputs, such as graphs, tables and dashboards.

## Limitations

Neotree has been implemented in three hospitals in two countries, therefore our learning about its efficacy and user acceptance are limited to these settings only. Although local government approval and integration with national systems can take time, conversations are ongoing to expand Neotree both within the current countries in which we have implemented and also in other countries. For example, we have just commenced work to adapt Neotree’s clinical content and functionality for use in primary health centres. Another limitation of the Neotree system is that it is currently available in English only, which could pose language barrier in countries where English is not the primary language. This feature could be co-developed with local partners as we expand in other countries and regions. While the system is yet to be fully integrated with the national EHR systems in Zimbabwe and Malawi, this work is in progress as described below.

## Future directions

Based on successful implementation of Neotree in two sites in Zimbabwe, the team is working on integrating the platform with the Zimbabwean national EHR system. This is an important aspect to maintain sustainable implementation of such learning healthcare systems in LRS. Integration within the national healthcare system will ensure: a) Neotree continues to reach more healthcare partners in those countries and help save more newborn lives, b) The data collected using Neotree will be available alongside any other health-related data that are being collected systematically across different health facilities, which can be used to follow patient journeys and guide the healthcare of those babies as they grow into children and adults, and c) minimisation of the information technology overhead associated with managing Neotree. By aligning our technology with broader health systems and technologies being implemented in-country, we can make sure that the Neotree can be setup and supported by the people and infrastructure that are managing other health systems, minimising the incremental cost of running the Neotree system in environments which are very cost-constrained. We are also working towards a similar approach in Malawi.

Other areas of future development in both Malawi and Zimbabwe include: a) development or a perinatal care module: Mummytree to capture clinical and sociodemographic data from the mothers during birth and to provide clinical decision support during labour as these factors can have significant impact on neonatal health and outcomes and therefore we can direct appropriate care where needed, b) Capturing of daily births and outcomes rather than being limited to NICU admissions only, so that these records can be linked to other health records in the future; c) adaptation and rollout in primary care facilities since the implementation has focused on hospitals so far and 50% of mothers deliver in primary health clinics with outcomes no better than if they had experienced a home birth, and d) “filling in the gaps” to provide data capture and clinical decision support for the entire admission period. While some key challenges exist, such as, ongoing staffing, data quality management and adjusting the system to available resources, the Neotree team continues to work with and for HCPs to address these challenges and find innovative solutions as the system matures.

## Conclusions

New initiatives and systems to improve and standardise newborn care have been developed in recent years, driven by the SDG goals to reduce newborn deaths in low and middle-income countries. However, many of these isolated systems address data capture, education and clinical decision support as discrete issues ( Bucher *et al.,* 2020; Chan *et al.,* 2021; Muhindo *et al.,* 2021; Schaeffer *et al.,* 2019). While Neotree has incorporated learning from these isolated systems wherever possible, the key strength of the Neotree platform is in our integrated approach. The comprehensive management platform ensures every newborn benefits from education in newborn care and data-driven clinical advice without assuming prior knowledge.

Neotree has already helped with caring for over 21,000 babies and supporting education of HCPs to improve newborn care. By prospectively capturing data at the bedside as part of usual daily care, Neotree offers impact at the point-of-care and avoids duplication of labour as it is not dependent on retrospective data entry from paper records. Even though high-resource countries use digital CDS to optimise quality of care, these are largely not generalisable to LRS. Such settings require technological adjustments to ensure efficient data flow and reporting within the system without internet connectivity. To ensure applicability and adaptability in LRS, Neotree has been rigorously tested and developed in collaboration with local clinicians and technology developers. Systematic checks applied at the point of data entry and continuous monitoring and assessment by the users result in overall quality improvement of collected data, compared to paper-based data collection.

Other similar healthcare systems are often proprietary, siloed, and address a narrow range of newborn conditions and assume prior clinical knowledge in newborn care. In comparison, the open-source nature of Neotree means that it can be fully owned (both the technology system itself and the data created) locally, with no reliance on the vendor that developed the code, as well as no risk that the technology will be charged for in the future. Furthermore, the platform can be adapted to each specific clinical context, and for wider applications, without being disease specific. This allows addressing a broader range of conditions that are deemed important by HCPs in specific sites and countries. Similarly, newborn care educational programs have demonstrated improvements in knowledge but have not resulted in sustained changes in practice, and reliance on paper-based reporting has hampered implementation ( Chan *et al.,* 2021). Neotree incorporates existing guidance to support sustained changes in clinical practice, and has flexibility to allow amending of guidelines when needed.

As the Neotree system continues to mature through the ongoing cycle of implementation, evaluation and learning, we envisage this free, open-source system being integral to providing better newborn care in low-resource facilities across the world. By supporting bedside decision making and collecting better quality newborn data, Neotree can identify areas where further research and dedicated use of resources can help to reduce newborn deaths and thus support these countries to work towards meeting the UN SDG.

## Data Availability

Zenodo: Sample dataset - Neotree. https://doi.org/10.5281/zenodo.7380945 ( Khan, 2022) This project contains the following underlying data: dummy_dataset.csv Data are available under the terms of the Creative Commons Attribution 4.0 International license (CC-BY 4.0).

## Software availability

**Source code available from:** https://github.com/neotree/neotree

**Archived source code at the time of publication:** https://doi.org/10.5281/zenodo.7380936 ( Khan *et al.* 2022)

**License:** MIT
