# Supplementary material for: Software development process of Neotree - a data capture and decision support system to improve newborn healthcare in low-resource settings
Source: Wellcome Open Res. 2022 Dec 19;7:305. [Version 1] doi: 10.12688/wellcomeopenres.18423.1 (PMC10682609; doi:10.12688/wellcomeopenres.18423.1)
Supplement: Peer review history

# Reviewer report: Reviewer response for version 1

**DOI:** 10.21956/wellcomeopenres.20430.r69380

**Published:** 2023-11-27

**Authors:** Manoj Maharaj

**Affiliations:** 1 University of KwaZulu-Natal, Durban, South Africa

The paper presents an overview of the development and potential of Neotree, an innovative software tool designed to enhance neonatal care in low-resource environments.

 The lack of advanced digital health solutions in low-resource settings necessitated the creation of Neotree, a tool that integrates data capture with clinical decision support, aiming to provide real-time insights and guidance for healthcare professionals. This user-centred approach ensures the tool's practical utility and relevance to the intended users.

 The technical description of Neotree is robust, detailing the software's architecture. The open-source nature of the project and its iterative, user-centred design process enhance its credibility. The system's integration with clinical workflows and attention to data management and security aspects demonstrate a comprehensive understanding of the healthcare context. These factors collectively contribute to the tool's technical robustness. The paper's focus on user-centred design, iterative development, and integration strategies provides a solid foundation for replication in similar contexts.

 The Neotree software is a significant advancement in digital health, particularly for neonatal care in low-resource settings. Its development addresses a critical need for specialized tools in such environments. The paper is a valuable resource for future developments in this domain. The detailed account of Neotree's development process provides important insights for similar initiatives. Ongoing evaluation and feedback collection from end-users will be crucial in refining and improving the tool.

 The paper contributes significantly to the digital health landscape. Its focus on a user-centred design process, coupled with a clear understanding of the challenges in low-resource settings, makes it a vital tool for improving neonatal healthcare. The authors have provided a thorough and insightful account of Neotree's development, offering valuable guidance for future digital health initiatives. The paper successfully demonstrates the potential of digital solutions in transforming healthcare in challenging environments.

Are the conclusions about the tool and its performance adequately supported by the findings presented in the article?

Yes

Is the rationale for developing the new software tool clearly explained?

Yes

Is the description of the software tool technically sound?

Yes

Are sufficient details of the code, methods and analysis (if applicable) provided to allow replication of the software development and its use by others?

Yes

Is sufficient information provided to allow interpretation of the expected output datasets and any results generated using the tool?

Yes

Reviewer Expertise:

Information Systems Research, ICT4D

I confirm that I have read this submission and believe that I have an appropriate level of expertise to confirm that it is of an acceptable scientific standard.

# Reviewer report: Reviewer response for version 1

**DOI:** 10.21956/wellcomeopenres.20430.r66465

**Published:** 2023-10-13

**Authors:** Katherine Kalaris

**Affiliations:** 1 University of Oxford, Oxford, England, UK

This article provides an overview of the development of a digital health intervention for newborn care- Neotree. Neotree includes clinical decision making support, data collection, and data dashboard to view performance. Development of Neotree has progressively occurred over eight years in collaboration with hospitals in Zimbabwe and Malawi and the authors describe in detail the development process and future directions for the tool.  

 Introduction: One of the reason for persistent high mortality despite facility deliveries is poor quality of care, commenting on this would strengthen the introduction. I would also suggest adding the newborn mortality rate for central and southern Asia when it is referenced in comparison to SSA.

 Conceptualisation: Do you know why the existing interventions were not being used? What did you learn from them to do differently with Neotree?

Are the conclusions about the tool and its performance adequately supported by the findings presented in the article?

Yes

Is the rationale for developing the new software tool clearly explained?

Yes

Is the description of the software tool technically sound?

Yes

Are sufficient details of the code, methods and analysis (if applicable) provided to allow replication of the software development and its use by others?

Yes

Is sufficient information provided to allow interpretation of the expected output datasets and any results generated using the tool?

Yes

Reviewer Expertise:

Health systems; MNH

I confirm that I have read this submission and believe that I have an appropriate level of expertise to confirm that it is of an acceptable scientific standard.
